# Non-Classical Gluconeogenesis-Dependent Glucose Metabolism in *Rhipicephalus microplus* Embryonic Cell Line BME26

**DOI:** 10.3390/ijms16011821

**Published:** 2015-01-14

**Authors:** Renato Martins da Silva, Bárbara Della Noce, Camila Fernanda Waltero, Evenilton Pessoa Costa, Leonardo Araujo de Abreu, Naftaly Wang’ombe Githaka, Jorge Moraes, Helga Fernandes Gomes, Satoru Konnai, Itabajara da Silva Vaz, Kazuhiko Ohashi, Carlos Logullo

**Affiliations:** 1 Laboratory of Chemistry and Function of Proteins and Peptides, Animal Experimentation Unit, UENF, Av. Alberto Lamego, 2000, Horto, CEP 28013-602 Campos dos Goytacazes, RJ, Brazil; E-Mails: rjrenato@ig.com.br (R.M.S.); babifal@yahoo.com.br (B.D.N.); kmhilaf@hotmail.com (C.F.W.); eveniltonpessoa@yahoo.com.br (E.P.C.); leoabreu@gmail.com (L.A.A.); 2 Laboratory of Infectious Diseases, Graduate School of Veterinary Medicine, Hokkaido University, Kita 18, Kita-ku Sapporo 060-0818, Japan; E-Mails: naftalygitahi@yahoo.com (N.W.G.); konnai@vetmed.hokudai.ac.jp (S.K.); kazu@chem.kyushu-univ.jp (K.O.); 3 Laboratory of Biochemistry Hatisaburo Masuda, Institute of Medical Biochemistry, Federal University of Rio de Janeiro, NUPEM-UFRJ/Macaé, Campus Macaé, Avenida São José do Barreto, São José do Barreto, CEP 27965-045 Macaé, RJ, Brazil; E-Mails: jlcmoraes@yahoo.com.br (J.M.); hgomes2@yahoo.com.br (H.F.G.); 4 Center of Biotechnology, Federal University of Rio Grande do Sul, C.P. 15005, Av. Bento Gonçalves 9500, Prédio 43421, Campos do Vale, CEP 91501-970 Porto Alegre, RS, Brazil; E-Mail: itabajara.vaz@ufrgs.br

**Keywords:** metabolism, gluconeogenesis, glycolysis, tick, gene expression, glucose

## Abstract

In this work we evaluated several genes involved in gluconeogenesis, glycolysis and glycogen metabolism, the major pathways for carbohydrate catabolism and anabolism, in the BME26 *Rhipicephalus microplus* embryonic cell line. Genetic and catalytic control of the genes and enzymes associated with these pathways are modulated by alterations in energy resource availability (primarily glucose). BME26 cells in media were investigated using three different glucose concentrations, and changes in the transcription levels of target genes in response to carbohydrate utilization were assessed. The results indicate that several genes, such as *glycogen synthase* (*GS*), *glycogen synthase kinase 3* (*GSK3*), *phosphoenolpyruvate carboxykinase* (*PEPCK*), and* glucose-6 phosphatase* (*GP*) displayed mutual regulation in response to glucose treatment. Surprisingly, the transcription of gluconeogenic enzymes was found to increase alongside that of glycolytic enzymes, especially pyruvate kinase, with high glucose treatment. In addition, RNAi data from this study revealed that the transcription of gluconeogenic genes in BME26 cells is controlled by GSK-3. Collectively, these results improve our understanding of how glucose metabolism is regulated at the genetic level in tick cells.

## 1. Introduction

The cattle tick *Rhipicephalus microplus* is an ectoparasite found in tropical and subtropical regions. Its importance in veterinary lies in its ability to transmit pathogens. It causes considerable losses in the cattle industry, with substantial damage to livestock [1]. Together, the economic losses caused by *R. microplus* parasitism and costs associated with its control in Brazil are estimated at 3 billion U.S. dollars a year [2].

Currently, few studies have investigated the mechanisms underlying energy metabolism during embryonic development in *R. microplus* or in the BME26 tick cell line [3]. Recent works have provided some insights into the dynamic processes that accompany nutrient utilization during tick embryogenesis [4,5,6].

Embryogenesis has been classically described as an energy-consuming process [7,8]. For oviparous organisms, the embryonic stage is characterized by the mobilization of metabolites of maternal origin for the development of new tissues and organs [9]. Studying the molecules involved in metabolic pathways during embryogenesis could reveal regulatory networks that control metabolism during embryonic development in numerous organism species. However, despite the recent advancements in molecular information, our understanding of genetic regulatory mechanisms, including that controlling energy metabolism, remains incomplete. In fact, many relevant aspects of metabolism during embryogenesis are not studied to the appropriate extent at present; however, essential pathways, such as those related to carbohydrate metabolism, are likely to be highly conserved among important disease vectors, including ticks and mites.

During embryogenesis, before blastoderm formation (a landmark stage of tick embryonic development), glycogen reserves are preferentially mobilized to support the energy-intensive process of embryogenesis [5]. Subsequently, protein degradation and gluconeogenesis intensify, in order to supply the embryo with sufficient glucose to allow glycogen resynthesis. Thus, the use of amino acids as a substrate for gluconeogenesis and the subsequent glycogen resynthesis play an important role during the stages of *R. microplus* embryogenesis. Glycogen is the main energy source during the early stages of *R. microplus* embryogenesis, and protein degradation increases during late embryogenesis [5]. Protein metabolism depends strongly on the substantial expression and activity of carbohydrate metabolism enzymes. The opposite is true for *Aedes aegypti* mosquitoes, with glycogen and protein levels decreasing 24 h into embryonic development, with a concomitant increase in the activity of phosphoenolpyruvate carboxykinase (PEPCK), a key gluconeogenic enzyme [10]. Thus, energy homeostasis is maintained by glycogen and protein mobilization at the end of mosquito embryonic development. However, the molecular mechanisms that regulate this process are poorly understood at present. Previous work by our group investigated the insulin-signaling pathway (ISP) and its possible role during embryogenesis, using the BME26 cell line as a model [3]. Compared with untreated cells, exogenous insulin elevated the cell glycogen content in the absence of fetal calf serum (FCS). Moreover, in the presence of PI3K inhibitors (wortmannin or LY294002), these effects were blocked. These results strongly suggested the presence of an insulin-responsive system in BME26 cells that may correlate with carbohydrate/glycogen metabolism during embryogenesis. GSK3 knockdown in *R. microplus* females resulted in a strong reduction in GSK-3 expression in ovaries, followed by significant reductions in both oviposition and hatching [11]. Moreover, similar effects were observed in females treated with GSK3 inhibitors (alsterpaullone, bromo-indirubin-oxime-6, and indirubin-3-oxime). The appearance of the eggs also changed with these treatments, suggesting an important role for GSK3 in proper embryonic development. Another recent study reported that monoclonal antibodies for triosephosphate isomerase (TIM) inhibited BME26 cell growth [6], providing further evidence of the importance of glucose metabolism in cell proliferation. However, few studies have addressed the molecular mechanisms that control the expression of genes that are central to energy metabolism. Our previous works focused on distinct protein targets involved in tick energy metabolism, with the aim of improving our understanding of tick physiology.

BME26 cells were initially characterized by Esteves* et al.* [12]. Since then, BME26 cells have been used to examine regulators of glycogen metabolism under experimental conditions [3,12]. The objective of the present study was to investigate the transcriptional profiles of important genes involved in energy metabolism in BME26 cells cultured under three different conditions: (i) cells treated with a high glucose concentration (100 mM); (ii) cells treated with a low glucose concentration (without additional glucose); and (iii) cells maintained under standard glucose concentration (50 mM) that is used in BME26 maintenance media (control cells). Glycolysis, gluconeogenesis, glycogenolysis and glycogen synthesis pathways have been described in other organisms [4,5,10]. Research about these genes is important for the understanding of genetic causes of flux through these pathways in tick cells, with the aim of further elucidating arthropod physiology. In this regard, the development of tick cell cultures has presented great opportunities for performing experiments under controlled conditions, interfering with metabolic pathways, and understanding the processes underlying genetic regulatory networks, metabolic fluxes, and the regulation of energy homeostasis. Thus, characterizing the expression patterns of key enzymes in energy metabolism in ticks [4,5,12] may yield new targets for developing novel acaricides and other interventions to control *R. microplus* infestations.

## 2. Results and Discussion

**Figure 1:**
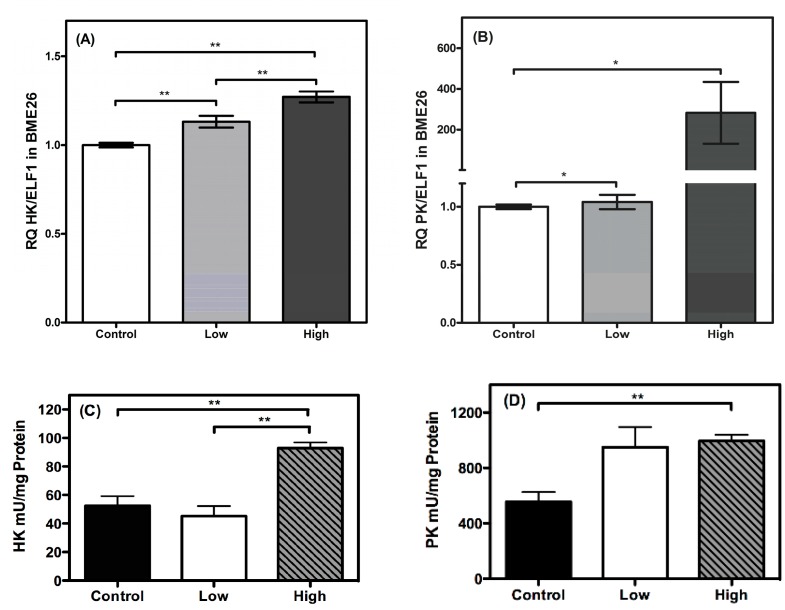
Transcriptional and activities of glycolytic enzymes are glucose concentration-dependent in BME26 cells. Transcriptional analysis of *hexokinase* (**A**); *pyruvate kinase* (**B**); HK activity (**C**) and pyruvate kinase activity (**D**), glycolytic key-enzymes, in embryonic *Rhipicephalus microplus* cells (BME26) in response to glucose treatment. Control: cells maintained with 50 mM of glucose; Low: cell maintained without glucose addition; and High: cells maintained with 100 mM of glucose. The experiment was performed with three independent biological samples in three experimental replicates each (* *p* < 0.05; ** *p* < 0.001, ANOVA).

Ticks are adapted to survive under different environmental conditions, including periods of low energy availability, e.g., during starvation [13,14]. These and other studies have suggested that alternative metabolic strategies, some exclusive to ticks, may exist under different environmental conditions. In the present work, the transcription of genes that regulate energy metabolism in *R. microplus* was characterized using the BME26 embryonic cell line cultured in the presence of high or low glucose concentrations. These different glucose concentrations were selected based on the glucose necessary to maintain the cell line under normal condition (50 mM), duplicating this glucose amount (100 mM, called high-glucose cells) or not adding the usual glucose amount (remaining with 3.125 mM, called low-glucose). Alterations in glucose availability affect the expression profiles of genes that encode glycolytic/gluconeogenic enzymes, as well as genes involved in glycogen metabolism, and in catabolic and anabolic pathways. Glycolysis is classically considered the principal pathway for carbohydrate catabolism, and pyruvate kinase (PK) and hexokinase (HK) are key enzymes in regulating this pathway. In the present work, the transcription of PK and HK was significantly increased in cells treated with a high glucose concentration (Figure 1). In mammals, these transcriptional changes were observed in cells after high glucose treatment [15]. In the current case, it is likely that the glycolytic pathway in tick cells accelerates, in response to greater glucose availability in the cytoplasm, because key enzymes for carbohydrate metabolism were highly up-regulated. A large number of genes show high expression in response to high carbohydrate feeding, including PK and HK in cultured hepatocytes [16,17]. Both of these glycolytic enzymes exhibit increases in the amounts of mRNA and protein, showing a clear sensitivity to glucose levels [18,19]. This hypothesis of sharpest glycolysis is supported by our data, whereby an increase in PK activity was observed in cells under high glucose treatment (Figure 1D), compared with the other conditions. Another glycolytic regulatory limiting step is triose phosphate isomerase (TIM), which also participates in gluconeogenesis. The TIM transcription level remained unchanged under varying glucose treatments relative to the control (data not shown). Because TIM reciprocally regulates both the carbohydrate catabolic and anabolic pathways [20], changes in substrate availability in these pathways probably do not alter TIM transcription, unlike other key enzymes exclusive to the glycolysis pathway, such as HK and PK (Figure 1).

**Figure 2:**
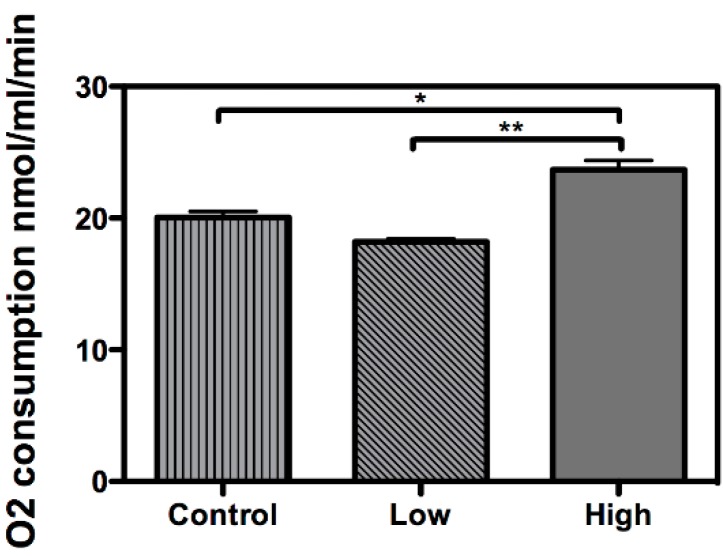
Higher oxygen consumption under high-glucose treatment. The oxygen consumption rate was measured in embryonic *Rhipicephalus microplus* cells (BME26) in response to glucose treatment. Control: cells maintained with 50 mM of glucose; Low: cell maintained without glucose addition; and High: cells maintained with 100 mM of glucose. The experiment was performed with three independent biological samples in three experimental replicates each (* *p* < 0.05; ** *p* < 0.001, ANOVA).

Interestingly, HK exhibited a higher relative transcription level in the cells treated with a low glucose concentration (Figure 1A), while HK activity remained unaltered during the same treatment (Figure 1C), underlining a differential relationship between transcriptional and enzymatic regulation for this enzyme. HK is responsible for phosphorylating glucose at carbon 6 to generate glucose-6-phosphate, allowing it to be trapped inside the cell and to be channeled into the glycolytic pathway, the pentose phosphate pathway, or glycogen synthesis, and relies on a refined allosteric regulation mechanism [21]. Thus, HK activity possibly will present a different response of the transcriptional rate. In the presence of high glucose concentration, HK activity and transcription increased, as observed for PK. Our data also shows a higher oxygen consumption during high glucose treatment (Figure 2), suggesting (in addition with HK and PK) an increased level of glycolysis specific to this condition, not with the low glucose treatment.

**Figure 3:**
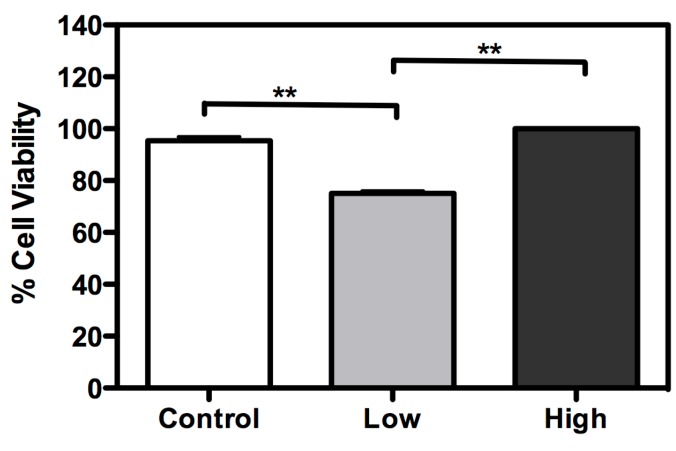
Glucose availability has an essential role in BME26 cell survival. Cell viability was performed in embryonic *Rhipicephalus microplus* cells (BME26) in response to glucose treatment. Control: cells maintained with 50 mM of glucose; Low: cell maintained without glucose addition; and High: cells maintained with 100 mM of glucose. The experiment was performed with three independent biological samples in three experimental replicates each (** *p* < 0.001, ANOVA).

**Figure 4:**
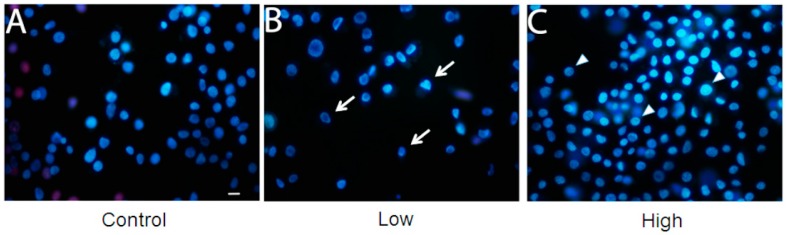
Membrane integrity is unaffected in BME26 cells after glucose treatment. The cells were directly stained by adding Hoechst 33342 and propidium iodide. Glass slides were observed in a fluorescence microscope (model Eclipse 80i, Nikon), and pictures were obtained at 400× magnification. Control: cells maintained with 50 mM of glucose (**A**); Low: cell maintained without glucose addition (**B**); and High: cells maintained with 100 mM of glucose (**C**). The arrows indicate the shape of low-glucose cells and the triangles indicate the rounded shape of high-glucose cells. Scale bar: 10 μm.

A cell viability assay was performed for the three culture conditions (Figure 3). With low glucose treatment, cell viability was lower than with other treatments. However, cell viability was enhanced with treatment with a high glucose concentration, when compared to control (cells maintained with a usual glucose concentration). Mitochondrial hexokinase activity is critical for sustaining constant ADP steady-state cycling, which in turn reduces the membrane potential and consequently decreases mitochondrial ROS formation, as previously described in rat brain cells [22,23]. Thus, glucose supports an increase in HK activity, leading to oxidative stress protection and higher cell survival [23]. Furthermore, if glucose availability is extremely high, the mitochondrial hexokinase activity decreases when ADP is produced. In this case, ROS production leads to a decrease in cell viability [24]. In this study, an increase in glucose disposal under high glucose condition is likely to improve the cell’s energetic fitness, resulting in higher viability, unlike cells cultured in low glucose levels. Furthermore, the microscopy analysis with propidium iodide did not show loss of membrane integrity, in any treatment (Figure 4).

The regulation of glycogen synthesis and degradation is a complex process that involves several enzymes and control points, and it is highly sensitive to changes in cell energy balance [25,26]. When the insulin-signaling pathway (ISP) is activated in response to elevated glucose, GSK3 activity is reduced and GS phosphorylation (inhibition) is blocked [27,28]. Under this condition, GS remains active and catalyzes glycogen synthesis. In BME26 cells, it was recently demonstrated that ISP was activated in response to exogenous insulin with increased glycogen levels [3], and these effects were cancelled out in the presence of PI3K inhibitors (wortmannin or LY294002). However, a transcriptional analysis of the genes involved in glycogen synthesis and degradation has not been reported in tick cells.

**Figure 5:**
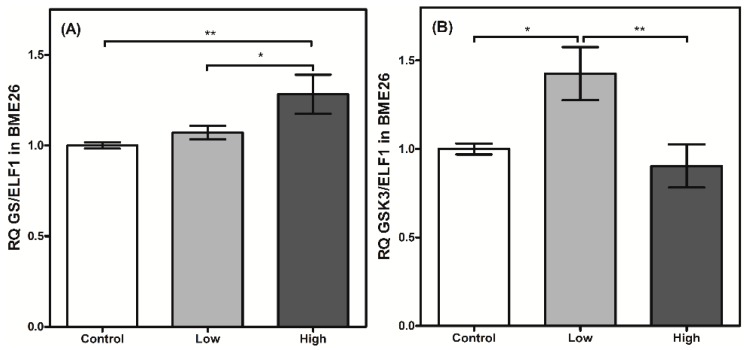
Glycogen metabolism synthesis has a transcriptional control in BME26 cells. Transcriptional analysis of *glycogen synthase* (**A**) and *glycogen synthase kinase 3* (**B**) in embryonic *Rhipicephalus microplus* cells (BME26) in response to glucose treatment. Control: cells maintained with 50 mM of glucose; Low: cell maintained without glucose addition; and High: cells maintained with 100 mM of glucose. The experiment was performed with three independent biological samples in three experimental replicates each (* *p* < 0.05; ** *p* < 0.001, ANOVA).

GSK3 transcription was elevated in the cells treated with low glucose concentrations in the present study. This enzyme participates in numerous cellular processes, including the regulation of GS by phosphorylation [27]. Apparently, a higher transcriptional level of GSK3 did not affect GS transcription in the low-glucose cells (Figure 5), reinforcing the reciprocal regulation between both enzymes that occurs at the enzymatic level. However, when the cells were treated with high amounts of glucose, GSK3 transcription was significantly reduced (Figure 5B), accompanied by an increase in GS transcription in cells under similar treatment (Figure 5A). This result may suggest the presence of mutual transcriptional regulation. Metabolic pathways that respond to cell glucose availability usually exhibit mutual transcriptional regulation [29]. In cells stimulated by insulin, AKT induces HK/GK expression [30]. A similar process is observed between PFK2/FBPase2 and HK/GK [31]. Nevertheless, high-glucose treatment can lead to increased levels of glycogen, which is normally stored under these conditions [3]. Therefore, this higher GS transcription could be directly related to an increase in the amount of glucose entering the cell, and may not be related to the reduction in GSK3 transcription itself, because the supposedly coordinated regulation between both enzymes was not observed in all the glucose treatments. Recently, Abreu* et al.* [32] reported that the GSK3/ATK axis in BME26 cells is involved in glycogen synthesis regulation and cell survival. AKT is a component of ISP, and phosphorylates a wide range of substrates, including GSK3, resulting in GSK3 inhibition [33,34]. AKT transcription did not change 24 h after the glucose treatments (data not shown). Collectively, the transcription of these enzymes appears to respond to changes in glucose availability and may share a mutual genetic regulation.

**Figure 6:**
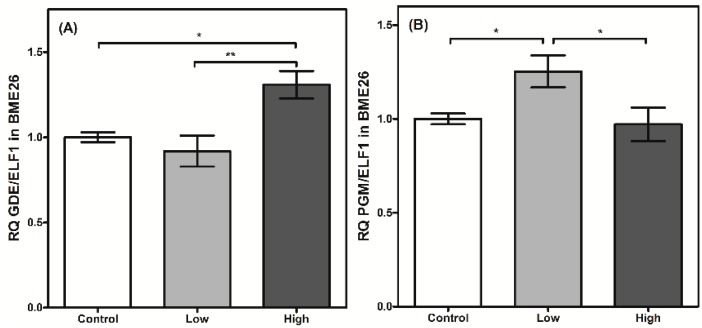
Glycogen degradation has a transcriptional control in BME26 cells. Transcriptional analysis of *glycogen debranching enzyme* (**A**) and *phosphoglucomutase* (**B**) in embryonic *Rhipicephalus microplus* cells (BME26) in response to glucose treatment. Control: cells maintained with 50 mM of glucose; Low: cell maintained without glucose addition; and High: cells maintained with 100 mM of glucose. The experiment was performed with three independent biological samples in three experimental replicates each (* *p* < 0.05; ** *p* < 0.001, ANOVA).

The regulation of glycogen mobilization was assessed by analyzing PGM and GDE transcription. Both enzymes are involved in glycogen polymer degradation. GDE cleaves the α-1,6-glycosidic bond between adjacent glucose molecules in the glycogen polymer, assisting glycogen phosphorylase and PGM [35,36]. PGM transcription is elevated in low-glucose cells (Figure 6B). This result suggests a type of genetic regulation that increases the transcript level of PGM when glucose availability is low and glycogen mobilization is necessary. On the other hand, the transcription of GDE does not change when glucose amounts are low (Figure 6A). However, when the cells were treated under high-glucose conditions, GDE transcription increased significantly, indicating a differential genetic regulation in relation to PGM.

**Figure 7:**
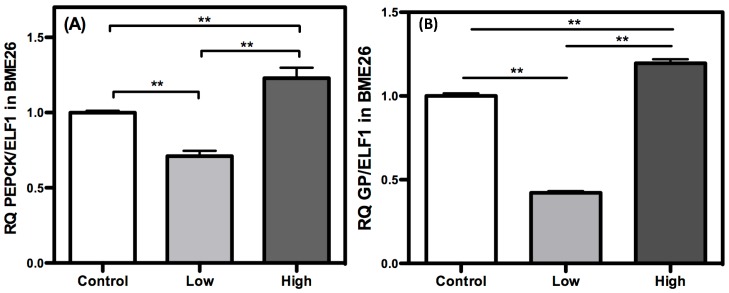
Gluconeogenic response increase in high glucose concentration in BME26 cells. Transcriptional analysis of PEPCK (**A**) and *glucose-6-phosphatase* (**B**), gluconeogenic key-enzymes, in embryonic *Rhipicephalus microplus* cells (BME26) in response to glucose treatment. Control: cells maintained with 50 mM of glucose; Low: cell maintained without glucose addition; and High: cells maintained with 100 mM of glucose. The experiment was performed with three independent biological samples in three experimental replicates each (** *p* < 0.001, ANOVA).

Gluconeogenesis produces glucose from non-glycosidic compounds, and it is an important strategy for the maintenance of cell energy homeostasis. PEPCK and GP are regulatory enzymes that catalyze the initial and final steps of gluconeogenesis, respectively [37,38]. GP removes a phosphate from glucose-6-phosphate to produce free glucose. Both enzymes exhibited similar transcriptional profiles across glucose treatments, showing reduced amounts of transcripts in the low glucose-treated cells (Figure 7). In the high-glucose cells, the opposite was observed, with increased transcription levels of both PEPCK and GP. Under normal physiological conditions, when glucose levels become low, as in starvation, the gluconeogenic flux accelerates [39,40]. In such cases, it is expected that these enzymes will undergo higher transcription when glucose levels are reduced, particularly PEPCK, as an enzyme that is mainly regulated by transcription in mammals. Surprisingly, in BME26 cells, this transcriptional profile was reversed. In cell culture, because there are no groups of specialized cells as seen* in vivo*, glucose dephosphorylation catalyzed by GP would result in the release of glucose and, consequently, the loss of this metabolite. Cells treated with low glucose may lack carbohydrate reserves [15], and a decrease in GP transcription is necessary to avoid the additional loss of glucose content. On the other hand, GP transcription increases in high-glucose cells. Massillon [41] observed an increase in GP transcription when glucose levels were elevated in hepatocyte cell culture, showing a dose-dependent response. The uptake of large amounts of glucose and the consequent phosphorylation of glucose inhibits HK activity as a result of elevated glucose-6-phosphate levels, leading to ROS generation by mitochondria [22]. In the context of cell culture, GP may be necessary to allow the diffusion of excess glucose away to avoid cell damage. PEPCK is also increased at the transcriptional level in high glucose-treated cells (Figure 7A). In addition to regulating gluconeogenesis, PEPCK regulates glyceroneogenesis, a pathway required for free fatty acid re-esterification to maintain an active level of triglyceride synthesis [38,42]. Under high carbohydrate availability, the flux through glyceroneogenesis increases [38]. Due to this phenomenon, we postulated that an increase in PEPCK transcription reflects a condition of high energy availability and possible glyceroneogenesis induction. Such genic profile in high glucose tick cells is very similar to observed in mammalian diabetic cells [43]. Interestingly, the glycolysis presented increased at this same moment, suggesting a deviation of pyruvate produced by glycolysis to gluconeogenesis or glyceroneogenesis (Figure 1). Indeed, a change in cell phenotype characterized by altered morphology was observed following glucose treatments. Cell culture heterogeneity was higher in the low-glucose cells than in control cells.

**Figure 8:**
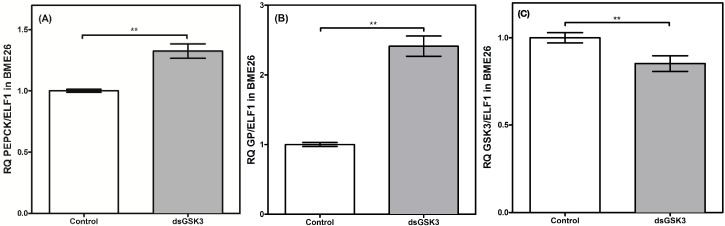
Gluconeogenic enzymes have transcriptional control by GSK3 in BME26 silenced-cells. Transcriptional analysis of PEPCK (**A**) and glucose-6 phosphatase (**B**), gluconeogenic key-enzymes, in embryonic *Rhipicephalus microplus* cells (BME26) in response to GSK3 silencing (**C**). Control: cells maintained with 50 mM of glucose; Low: cell maintained without glucose addition; and High: cells maintained with 100 mM of glucose. The experiment was performed with three independent biological samples in three experimental replicates each (** *p* < 0.001, paired T test).

These results demonstrate a differential genetic regulation between cell culture and* in vivo* models. Nevertheless, both gluconeogenesis enzymes were regulated in a coordinated manner, with similar transcriptional profiles. Our group has been investigating the role of GSK3 in a wide range of metabolic processes, including gluconeogenesis. GSK3 knockdown induces increases in PEPCK and GP transcription levels (Figure 8), suggesting an indirect genetic regulation of the gluconeogenesis pathway through GSK3. A high silencing rate of essential metabolic genes usually leads to cell lethality. Specifically, a GSK3 knockdown (around 90%) in female mosquito *Aedes fluviatilis* prevented them from developing their ovaries as well as egg laying. So, in a new experiment, GSK3 transcription was reduced by 30% to evaluate the effect of GSK3 inhibition on insect embryogenesis [44]. Similarly, we induced a higher efficiency in GSK3 silencing in the BME26 cells previously, leading to cell death (data not shown). Thus, a methodology was adopted to interfere in the transcriptional response, but without inducing cell death. At enzymatic level, GSK3 is regulated by phosphorylation, and a 15% of suppression may not affect the metabolism overall substantially. Effectively, the low rate in GSK3 silencing afforded to analyze the effect of GSK3 reductions in the transcription of gluconeogenesis genes.

**Figure 9:**
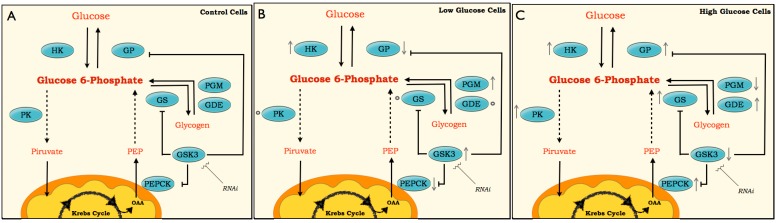
Scheme for pathways of glucose metabolism. The scheme is based on enzyme activities, metabolites and molecular changes in BME26 cells after glucose treatments. The enzymes studied are presented inside blue spheres (HK, PK, GS, GSK3, PGM, GDE, PEPCK and GP). Arrows next to the enzymes (↓↑) represent the oscillations in enzymatic activity and transcriptional response. Small spheres next enzymes (○) indicate no variations in these enzymes or genes compared with the control. Dashed line involves several enzymes steps in the pathway, and solid lid represents one enzymatic step. The low-glucose (**B**) and high-glucose (**C**) are compared with the control (**A**).

To date, and to our knowledge, no mutual regulation between GSK3 and gluconeogenesis has been reported. Future studies could focus on whether there is any direct enzymatic regulation between GSK3 and other gluconeogenic enzymes. Figure 9 summarizes all metabolic and molecular changes in the respective treatments. Altogether, these results contribute to the understanding of the mechanisms that control glucose metabolism at the genetic level in the model cell line BME26. Characterizing changes in metabolic enzymes at both the transcriptional and functional levels in response to nutrient availability may lead to the identification of genes that are critical for maintaining the cellular energy balance. Moreover, this research may yield potential antigens for improved anti-tick vaccines or novel targets for acaricide action, which are urgently needed to control the tick vector *R. microplus*.

## 3. Methods

### 3.1. BME26 Cell Line

Cells were maintained as previously described [12]. The BME26 cells were maintained in Leibovitz L-15 medium (Gibco BRL, Grand Island, NY, USA) supplemented with amino acids, glucose, mineral salts, and vitamins [45]. During medium preparation, glucose was added at two different concentrations, 50 mM (control-the usual glucose concentration) and 100 mM (high-glucose treatment). The medium was diluted in sterile water (3:1), and then tryptose phosphate broth (10%), fetal calf serum (10%), and penicillin/streptomycin (100 U/mL and 100 µg/mL, respectively) were added. Cells from confluent flasks (25 cm^2^) were resuspended in fresh complete medium using a 22-gauge needle with bent tip attached to a 5-mL plastic syringe. Culture density was determined using a Neubauer hemocytometer, and cell viability was determined using the trypan blue exclusion technique (0.4%, Sigma, St. Louis, MO, USA). An aliquot of 1 × 10^7^ viable cells was transferred to 5 mL (final volume) of fresh complete medium and incubated at 34 °C for up to two weeks to promote cell proliferation. The medium was replaced weekly to achieve high cell homogeneity. Next, 24-well plates (5 × 10^5^ cells/well) were seeded with cells suspended in 500 µL of normal medium, incubated overnight for adhesion. Then, normal medium was completely replaced by either medium without the addition of glucose 50 mM (remaining only with the glucose present in the medium, called low-glucose treatment), with a normal glucose concentration (control), or by a high glucose concentration (high-glucose treatment). In the low-glucose medium, the content of glucose is that present in the medium itself (3.125 mM) (Leibovitz Medium L-15 composition, number L4386-Sigma). All these glucose concentrations were chosen based on the glucose present in the control medium, duplicating this amount or without glucose addition.

### 3.2. Viability Assay

Cell viability was determined 24 h after treatment with different media using the MTT assay. Briefly, 50 µL MTT (5 mg/mL in PBS) were added on each well. After 2 h of incubation at 34 °C, the medium was completely discarded, and 1 mL of acid-isopropyl alcohol (0.15% HCl in isopropyl alcohol) was added to dissolve formazan crystals. The mixture was transferred to 1.5-mL tubes and centrifuged at 6000× *g* for 15 min, and the clear supernatant was collected for absorbance measurements at 570 nm in a UVmini-1240 UV–Vis spectrophotometer Shimadzu (Kyoto, Japan). Unless stated otherwise, the absorbance values of the control treatment were used for normalization (100% viability).

### 3.3. Membrane Integrity Analysis

BME26 cells were plated (3 × 10^5^ cells/well) onto glass coverslips placed at the bottom of a 24-well plate. The cells were treated with different glucose concentrations. Subsequently, the cells were directly stained by adding Hoechst 33342 (0.4 μg/mL, final concentration) for 5 min, followed by staining with propidium iodide (2 μg/mL, final concentration) for further 2 min. The loss of membrane integrity was detected in cells stained with Hoechst 33342/Propidium iodide observed with pink nuclei under fluorescence microscope (Nikon 80i). Hoechst 33342 permeable cells were stained with blue nuclei. The incubations were performed at room temperature and in the dark. The well contents were discarded, and the coverslips were washed with PBS before being mounted over glass slides with 5 μL of glycerol. Cells were observed under a fluorescence microscope (model Eclipse 80i, Nikon, Kanagawa, Japan), and images were obtained at 400× magnification.

### 3.4. Double-Stranded RNA (dsRNA) Synthesis

Oligonucleotide primers containing T7 promoter sequence were synthesized for the* in vitro* transcription and synthesis of dsRNA using RiboMAX™ Express RNAi System Kit (Promega, Madison, WI, USA). cDNA from 1-day-old tick eggs was used for dsRNA synthesis. The dsRNA was purified according to the manufacturer’s instructions, and its concentration was measured at 260 nm. An aliquot of dsRNA was analyzed by agarose gel electrophoresis to check for any degradation. Double-stranded RmGSK3 RNA was synthesized as described previously [12]. The negative control for the RNAi-induced gene silencing was an unrelated dsRNA designed for *E. coli* β-galactosidase (kindly donated by Professor Marcos H. Sorgine, Instituto de Bioquímica Médica (IBqM), Universidade Federal do Rio de Janeiro (UFRJ)). The sizes of the synthesized double-stranded RNA complexes were 635 bp for dsAKT, 800 bp for dsCN, and 798 bp for dsGSK.

### 3.5. dsRNA Delivery into BME26 Cells

BME26 cell suspensions were seeded into 24-well plates (5 × 10^5^ cells/well) to a final volume of 500 μL of complete medium. After 24 h of incubation at 34 °C, the culture medium was replaced with 200 μL of fresh medium containing 4.8 μg of dsRNA/well, with gentle mixing. Cells were incubated for an additional 24 h and were harvested for subsequent processing.

### 3.6. RNA Extraction

Total RNA was extracted from the BME26 cells harvested from 24-well plates using Trizol reagent (Invitrogen, Grand Island, NY, USA) according to the manufacturer’s instructions. One microgram of total RNA was reverse transcribed with the High-Capacity cDNA Reverse Transcription-M-MLV kit (Takara Biotechnology, Shiga, Japan). Amplifications were performed on the LightCycler platform (Roche, Göttingen, Germany). Serial dilutions of the cDNA were used to construct a calibration curve. Reaction efficiencies between 85% and 100% were determined from calibration curves for each set of primers in 10 μL reactions. The *R. microplus* elongation factor-alpha gene (Elf1A) was utilized as reference [46] to normalize the reactions. cDNA from the control BME26 cells was used as a calibrator for the assays. The relative expression of the calibrators was assigned a value of 1 unit. Statistical analyses (means and standard deviation) were performed on data from three independent experiments.

### 3.7. Relative Quantification of Metabolism Genes by Real-Time PCR

A relative transcriptional analysis [47] was conducted with cDNA as a template for quantitative PCR using the LightCycler 480 II platform (Roche, Mannheim, Germany). Serial dilutions of cDNA were used for calibration curve preparation. Reaction efficiencies between 85% and 100% were determined from the calibration curves for each set of primers in 10-μL reactions. The primers used to amplify the targets are listed in Table 1. The relative expression was determined using the Cp values from each run and the Relative Expression Software Tool [47].

Gene sequences for hexokinase (HK), *pyruvate kinase* (PK), *phosphoenolpyruvate carboxykinase* (PEPCK), *glucose 6-phosphatase* (GP), *glycogen synthase* (GS), *phosphoglucomutase* (PGM), *triose phosphate isomerase* (TIM), *protein kinase B* (AKT), *glycogen synthase kinase 3* (GSK3) and *glycogen-debranching enzyme* (GDE) from different species were retrieved from the GenBank database (Table 1) and used to conduct a BLAST search of an *R. microplus* transcriptome database generated using the Illumina Solexa sequencing platform (BioProject ID PRJNA232001 at Transcriptome Shotgun Assembly (TSA) database, GenBank). The *R. microplus* gene sequences for TIM, AKT and GSK3 were obtained from GenBank. Specific primers for the genes were designed based on these *R. microplus* sequences (see Table 1).

**Table 1:** The sequences of primers used for SYBR Green real-time PCR of genes involved in glycogen metabolism.

Gene	Primers Used for *R. microplus* Genes	Amplicon Size (bp)	Gene Used as Reference *	GenBank Access Number of *R. microplus* Gene
*Hexokinase*	CATGGACAAAGAGCTTCAACTGCTC GGAAAGCTCCCTTGACCAGGGTA	150	XM_002412706.1 *Ixodes scapularis*	KF951259
*Pyruvate kinase*	GGGCAAGAGGGCAAGACAACTG CACGTTGAGCACCTTGGTGATG	141	XM_002407420.1 *Ixodes scapularis*	KF951260
*Phophoenolpyruvate carboxykinase*	CAAGCAATGAGTGCCTGCCAC ACAGTCTTCCGTTTTCATCTTG	147	XM_002413329.1 *Ixodes scapularis*	KF951261
*Glucose-6-phosphatase*	GGCAGCCATTTGGTACATCATCC CGACAGGCTGACAATGCACAGG	133	XM_002407091.1 *Ixodes scapularis*	KF951262
*Glycogen synthase-6*	GCTGGTATCGGGCTGATCCTG GATGCCTCTGTCTCCAGCCTCC	165	XM_002435718.1 *Ixodes scapularis*	KF951264
*Phosphoglucomutase*	CGGATCTGGGCAAGCTGGG CCGTCGTGACCCTTGATGAGG	151	XM_003695907.1 *Apis florea*	KF951265
*Triose phosphate isomerizes*	CCTCGCTGCACAAAATTGCTAC TCCGAATGACCCAGTATGACCC	128		EF014474
*Protein kinase B*	GGCCAAAGCCATTCACCTTCA CCTCCTCACTCGCCAACTTCTC	151		JX648548
*Glycogen synthase kinase 3*	CCCACACCCGCTATTTATTG TGTGCAGGAGAGCCAGTTTA	113		EF142066
*Glycogen-debranching enzyme*	ATGCTCAGGATCACGCAGAAGC GTACGTCGGTTGGGAAGGACAAGG	173	XM_002401176.1 *Ixodes scapularis*	

* Access numbers of published DNA sequences used to conduct Blast searches for matching gene transcripts in the *R. microplus* database (PRJNA232001 TSA database).

### 3.8. Hexokinase (HK) Activity

BME26 cell suspensions were seeded into 24-well plates (5 × 10^5^ cells/well) to a final volume of 500 μL of complete medium and lysate with PBS (5 mg/mL). The cell lysate was assayed for HK activity in 20 mM Tris–HCl pH 7.5 containing 6 mM MgCl_2_, 1 mM ATP, 0.5 mM NAD^+^ and 10 mM NaF, and the reaction was started with 2 mM glucose. The glucose 6-phosphate that formed was measured by adding an equal volume of 20 mM Tris–HCl pH 7.5, 6 mM MgCl_2_, 1 unit/mL glucose 6-phosphate dehydrogenase from *Leuconostoc mesenteroides* and 0.3 mM β-NAD^+^. The production of β-NADH was determined at 340 nm using a molar extinction coefficient of 6.22 M^−1^, as previously described [48,49].

### 3.9. Pyruvate Kinase (PK) Activity

Samples were prepared as described in the HK activity section. PK activity was measured in 20 mM Tris–HCl pH 7.5, 5 mM MgCl_2_, 1 mM ADP, 0.4 mM NADH and 1 unit/mL lactate dehydrogenase, and the reaction was started with 1 mM PEP. The β-NADH consumption was evaluated using a Shimadzu U1240 spectrophotometer at 340 nm, with a molar extinction coefficient of 6.22 M^−1^, as previously described [48,49].

### 3.10. Oxygen Consumption

Total oxygen consumed by BME26 cells after glucose treatments were assayed using a Clark-type electrode (Yellow Springs Instruments Co., Yellow Springs, OH, USA). The calibration process was executed using 100% as complete air-saturated buffer at 28 °C. Measurements were carried out in 1.5 mL 20 mM PBS buffer (pH 7.4) and the rate of oxygen consumption was calculated in mmol O_2_/min/mL-cell. A solution containing 1 mM KCN was added to inhibit cytochrome oxidase. The same solution was used as negative control. Three assays were performed using 1 × 10^7^ cells/mL for three independent experiments in triplicate.

### 3.11. Statistical Analysis

The experiments were performed with three independent biological samples in three experimental replicates each, to obtain a mean. All data values were expressed as mean ± S.D. The ANOVA was used to determine significant differences between groups when data were normally distributed. The *t*-tests were used when comparing data between two groups (Figure 8). The Tukey test was used to compare data between three groups. Significance was set at * *p* < 0.05; ** *p* < 0.001, ANOVA).

## 4. Conclusions

In conclusion, a number of genes involved in the major pathways for carbohydrate catabolism and anabolism were evaluated in a tick embryonic cell line. The results show that *glycogen synthase* (*GS*), *glycogen synthase kinase 3* (*GSK3*), *phosphoenolpyruvate carboxykinase* (*PEPCK*) and *glucose 6-phosphatase* (*GP*), exhibited mutual regulation in response to glucose treatment. In this sense, both gluconeogenesis enzymes were regulated in a coordinated manner, with similar transcriptional profiles with the involvement of *GSK3*, indicating the correlation between glucose anabolism and catabolism control. Taken together, these results improve the understanding of glucose metabolism in ticks.

## Abbreviation

HK: hexokinase
PK: pyruvate kinase
GS: glycogen synthase
TIM: triose phosphate isomerase
AKT: protein kinase B
GSK3: glycogen synthase kinase 3
PGM: phosphoglucomutase
GDE: glycogen-debranching enzyme
PEPCK: phosphoenolpyruvate carboxykinase
GP: glucose-6 phosphatase
GK: glucokinase
